# Elevated cerebrospinal fluid 5-hydroxyindoleacetic acid in macaques following early life stress and inverse association with hippocampal volume: preliminary implications for serotonin-related function in mood and anxiety disorders

**DOI:** 10.3389/fnbeh.2014.00440

**Published:** 2014-12-23

**Authors:** Jeremy D. Coplan, Sasha L. Fulton, Wade Reiner, Andrea Jackowski, Venkatesh Panthangi, Tarique D. Perera, Jack M. Gorman, Yung-yu Huang, Cheuk Y. Tang, Patrick R. Hof, Arie Kaffman, Andrew J. Dwork, Sanjay J. Mathew, Joan Kaufman, J. John Mann

**Affiliations:** ^1^Nonhuman Primate Laboratory, Department of Psychiatry and Behavioral Sciences, Downstate Medical Center, State University of New YorkBrooklyn, NY, USA; ^2^Geriatric Psychiatry, New York State Psychiatric InstituteNew York, NY, USA; ^3^College of Medicine, State University of New York Downstate Medical CenterBrooklyn, NY, USA; ^4^Departamento de Psiquiatria & Neuroradiologia, Universidade Federal de São PauloSão Paulo, Brazil; ^5^Franklin Behavioral Health ConsultantsBronx, NY, USA; ^6^Department of Molecular Imaging and Neuropathology, New York State Psychiatric InstituteNew York, NY, USA; ^7^Departments of Psychiatry, Neuroscience, and Radiology, Icahn School of Medicine at Mount SinaiNew York, NY, USA; ^8^Fishberg Department of Neuroscience and Friedman Brain Institute, Icahn School of Medicine at Mount SinaiNew York, NY, USA; ^9^Department of Psychiatry, Yale University School of MedicineNew Haven, CT, USA; ^10^Mental Health Care Line, Michael E. Debakey VA Medical CenterHouston, TX, USA; ^11^Menninger Department of Psychiatry and Behavioral Sciences, Baylor College of MedicineHouston, TX, USA; ^12^Child Study Center, Yale University School of MedicineNew Haven, CT, USA

**Keywords:** variable foraging demand, MRI, cisternal tap, serotonin metabolite, monoamine metabolites

## Abstract

**Background:** Early life stress (ELS) is cited as a risk for mood and anxiety disorders, potentially through altered serotonin neurotransmission. We examined the effects of ELS, utilizing the variable foraging demand (VFD) macaque model, on adolescent monoamine metabolites. We sought to replicate an increase in cerebrospinal fluid (CSF) 5-hydroxyindoleacetic acid (5-HIAA) observed in two previous VFD cohorts. We hypothesized that elevated cisternal 5-HIAA was associated with reduced neurotrophic effects, conceivably due to excessive negative feedback at somatodendritic 5-HT_1A_ autoreceptors. A putatively decreased serotonin neurotransmission would be reflected by reductions in hippocampal volume and white matter (WM) fractional anisotropy (FA).

**Methods:** When infants were 2–6 months of age, bonnet macaque mothers were exposed to VFD. We employed cisternal CSF taps to measure monoamine metabolites in VFD (*N* = 22) and non-VFD (*N* = 14) offspring (mean age = 2.61 years). Metabolites were correlated with hippocampal volume obtained by MRI and WM FA by diffusion tensor imaging in young adulthood in 17 males [10 VFD (mean age = 4.57 years)].

**Results:** VFD subjects exhibited increased CSF 5-HIAA compared to non-VFD controls. An inverse correlation between right hippocampal volume and 5-HIAA was noted in VFD- but not controls. CSF HVA and MHPG correlated inversely with hippocampal volume only in VFD. CSF 5-HIAA correlated inversely with FA of the WM tracts of the anterior limb of the internal capsule (ALIC) only in VFD.

**Conclusions:** Elevated cisternal 5-HIAA in VFD may reflect increased dorsal raphe serotonin, potentially inducing excessive autoreceptor activation, inducing a putative serotonin deficit in terminal fields. Resultant reductions in neurotrophic activity are reflected by smaller right hippocampal volume. Convergent evidence of reduced neurotrophic activity in association with high CSF 5-HIAA in VFD was reflected by reduced FA of the ALIC.

## Introduction

Early life stress (ELS) increases the risk of the development of depression and anxiety disorders in adulthood (Heim and Nemeroff, 2001). Childhood trauma and neglect exerts a profound influence on behavior and physiology, putting victims at risk for depression, anxiety disorders and substance abuse (de Wilde et al., 1993; Sedlak and Broadhurst, 1996; Agid et al., 2000; Dube et al., 2003). Indeed, ELS may not only increase the risk for developing these disorders later in life, but may also precipitate the onset of these illnesses, increase the likelihood of comorbidity, and impair efficacy of treatment (Friedman et al., 2002; Nemeroff et al., 2003; Gladstone et al., 2004). Adverse early experiences may therefore confer susceptibility to psychiatric disorders in adulthood, in all likelihood, due to the detrimental effects of stressful events on neurodevelopment (Teicher et al., 2003; Watts-English et al., 2006). As the serotonergic system has been identified as crucially important to both the pathophysiology and the treatment of mood disorders, alterations in serotonin neurotransmission following ELS may be one key component in determining the contribution of ELS to depression and anxiety (Owens and Nemeroff, 1994). Although altered serotonin neurotransmission has been reported in subjects with ELS (Rosenblum et al., 1994; Kinnally et al., 2011), the consequences of ELS on serotonin neurotransmission are not well understood and require elucidation. Moreover, large scale studies indicate that less than 30% of patients with major depressive disorder (MDD) remit to the selective serotonin reuptake inhibitor (SSRI), citalopram (Trivedi et al., 2006) prompting renewed scrutiny of the serotonin system. For instance, ELS predicts poor response to treatment with the antidepressant, nefazadone (Nemeroff et al., 2003), suggesting modifications of the serotonin system that negate antidepressant efficacy.

In order to examine the pathophysiological effects of early life adversity, our laboratory has studied the variable foraging demand (VFD) model of ELS in bonnet macaques. In the VFD model, unpredictability of maternal food access compromises maternal care of the infant, producing an experimental homolog of human anxiety and mood disorders in the offspring (Rosenblum and Paully, 1984; Andrews and Rosenblum, 1988, 1991; Rosenblum et al., 1994). Although past research suggests that mood disorders may result from a serotonin deficit in stress-related brain regions, studies of CSF serotonin metabolite concentrations in patients with mood disorders have produced inconsistent results (Shaw et al., 1967; Bourne et al., 1968; Pare et al., 1969; Lloyd et al., 1974). Additional attempts to confirm reduced levels of the serotonin metabolite, 5-hydroxyindoleacetic acid (5-HIAA) in depressed subjects have been inconclusive (Bowers et al., 1969; Papeschi and McClure, 1971; Mendels and Frazer, 1974; Nordström and Åsberg, 1992; Mann et al., 1996a). Researchers have speculated that the inconsistency has been due to CSF 5-HIAA failing to detect localized serotonin deficits in brain sub-regions involved in mood disorders (Mendels and Frazer, 1974). Suicide victims have, however, consistently demonstrated reduced lumbar CSF 5-HIAA concentrations (Mann et al., 1996b). However, regional divergence in regional differences in serotonin and 5-HIAA in suicide victims (Bach et al., 2014) is discussed further below.

In two earlier studies, our laboratory found elevated concentrations of cisternal CSF 5-HIAA in VFD-reared vs. control subjects reared without maternal uncertainty (Coplan et al., 1998; Mathew et al., 2002). The finding of relatively higher concentrations of cisternal CSF 5-HIAA in VFD does not necessarily contradict the notion of a regional serotonin deficit in forebrain regions in mood disorders. We posit that the use of cisternal CSF taps preferentially accesses diffusion of 5-HIAA from the nearby midbrain serotonergic dorsal raphe (DR) region, thus potentially yielding a more accurate picture of localized monoamine activity than that provided by lumbar puncture. The latter conceivably samples an aggregate of serotonin metabolite levels from throughout the CNS, with putative mixing of high midbrain and low forebrain 5-HIAA, thereby obscuring regional differences (Bach et al., 2014). By contrast, a cisternal CSF 5-HIAA deficit is consistently observed in maternally-deprived peer-reared vs. maternally-reared rhesus monkeys (Higley et al., 1991), although it is important to note that the low CSF 5-HIAA associated with peer rearing is linked not only with affective disturbance but also with impulsivity and aggression (Higley and Linnoila, 1997). Using positron emission tomography, peer-reared subjects showed reduced serotonin transporter protein binding in both the dorsal raphe and hippocampus (Ichise et al., 2006), demonstrating that reduced CSF 5-HIAA is associated with deficits of serotonin in regions salient to the current study. Thus, maternal deprivation appears to ostensibly impact the serotonin system differently when compared to maternal unpredictability.

We hypothesized that the high cisternal CSF 5-HIAA found in VFD subjects therefore reflects a specific form of dysfunctional serotonin neurotransmission post-ELS. For instance, suicides are reported to have simultaneously hyperserotonergic midbrain raphe and hyposerotonergic prefrontal cortex (PFC) (Bach et al., 2014). The authors postulated stress-induced increase in tryptophan hydroxylase as the cause of increased brainstem 5-HIAA. PET studies by the same group suggest a greater number of autoreceptors in MDD as a cause of less serotonin neuronal firing and serotonin release (Parsey et al., 2006) but the hypothesis does not incorporate the observation of elevated midbrain 5-HIAA.

During infant neurodevelopment, adverse experiences cause persistent activation of stress-mediated circuits, sensitizing them to exhibit exaggerated responses to future stress (Heim and Nemeroff, 2001; Artigas et al., 2006). Previously identified glutamatergic DR afferents (Valentino et al., 2003) which arise in layer V of the medial prefrontal cortex, a stress-sensitive region (Celada et al., 2001), may induce heightened release of extracellular serotonin in the midbrain dorsal raphe region, activating a negative feedback mechanism mediated by 5-HT_1A_ autoreceptors. An alternate hypothesis invokes a developmental increase following ELS in midbrain tryptophan hydroxylase and 5-HIAA (Wilson et al., 2012). An overall reduction in serotonin neurotransmission to limbic structures such as the hippocampus, and other neurotrophic targets such as related white matter tracts, may ensue, independent of hypothesis (Celada et al., 2001; Valentino et al., 2003). We therefore surmise that high cisternal CSF 5-HIAA concentrations may be directly linked to an attenuation of serotonin outflow (Blier et al., 1998). Subsequently, this mechanism may represent an important facet of the increased vulnerability to stress and mood disorders following ELS (Heim and Nemeroff, 2001; Coplan et al., 2011).

In the current study we seek to replicate our finding of elevated CSF 5-HIAA in a third cohort of VFD animals. Furthermore, we sought to confirm our hypothesis that high cisternal CSF 5-HIAA is associated with a putatively reduced serotonin transmission from the DRN by first correlating the former with hippocampal volume. An inferred attenuation in serotonin output to the hippocampus would have a directly negative effect on serotonin intracellular messaging systems and neurotrophic activity. Diminished numbers of neurotrophic factors specifically affects neuronal growth and survival, leading to reduction in hippocampal volume (Duman et al., 1997, 2000). Dopamine and norepinephrine metabolites were also examined [homovanillic acid (HVA) and 3-methoxy-4-hydroxyphenylglycol respectively]. We have previously demonstrated elevated CSF HVA in VFD-reared subjects vs. non-VFD controls (Coplan et al., 1998), implicating dopamine as another monoamine system altered by early life stress. Although we failed to demonstrate rearing differences for CSF MHPG, we had previously reported altered sensitivity to the alpha-2 receptor antagonist, yohimbine, in VFD- vs. non-VFD reared subjects (Rosenblum et al., 1994). We wished to investigate whether there were alterations in the relationship between CSF MHPG and hippocampal volume following ELS, as reflective, in part, of serotonin-related alterations. We reported that in patients with panic disorder, enhanced serotonin neurotransmission induced by SSRI administration is associated with lowered plasma MHPG concentrations (Coplan et al., 1997). Serotonin attenuates neuronal response to glutamate, a major neurotransmitter utilized by afferents to the noradrenergic locus ceruleus (LC) (Aston-Jones et al., 1991). Reduced outflow of serotonin to target projections such as LC, resulting from elevations of peri-raphe serotonin, would favor CSF MHPG positively tracking CSF 5-HIAA. Thus, the relationship between CSF MHPG and hippocampal volume would be expected to mirror the relationship between CSF 5-HIAA and hippocampal volume.

Although less documentation exists, compromised serotonin neurotransmission may well influence the neurotrophic status of other brain regions in addition to the hippocampus. Alterations in white matter (WM) integrity as measured by FA have been observed throughout the brain across a range of psychiatric disorders (Daniels et al., 2013). Based on the notion that serotonin-dependent neurotrophic growth factors affect WM integrity measures (Martinowich and Lu, 2007), our own studies previously found FA reductions following ELS in the anterior limb of the internal capsule (ALIC), which lies in key circuitry connecting the temporal and frontal regions of the brain (Cannistraro et al., 2007; Zou et al., 2008). Although the uncinate fasciculus has featured prominently in studies of childhood maltreatment (Huang et al., 2012), and the fornix is closely related to the hippocampus (Kubicki et al., 2005), the ALIC shows decreased FA in neurodevelopmental disorders such as schizophrenia (Kubicki et al., 2005) and schizotypal personality disorders (Hazlett et al., 2012). Decreased FA of the ALIC has also been identified as a plausible link between type II diabetes and major depressive disorder (Zhang et al., 2013). In the present study, we examine the relationship between cisternal CSF 5-HIAA concentrations and FA measures derived from the ALIC. We hypothesized that high cisternal CSF 5-HIAA, specifically in VFD-reared subjects, would also be inversely associated with WM integrity in the ALIC, providing convergent evidence for serotonin-related neurotrophic compromise following ELS.

The primary hypothesis, therefore, was to detect elevated CSF 5-HIAA in VFD vs. non-VFD. The second hypothesis was to demonstrate an inverse correlation between CSF 5-HIAA concentrations and hippocampal volume in VFD, indirectly invoking a putative deficit in hippocampal serotonin neurotransmission. The third hypothesis was to demonstrate an inverse relationship between CSF 5-HIAA and FA of the ALIC in VFD, providing convergent evidence of neurotrophic compromise in serotonin target areas.

## Materials and methods

### Subjects

Twenty-two bonnet macaque (*Macaca radiata*) subjects reared under conditions of ELS (VFD) were compared to 14 subjects reared under normative conditions (non-VFD). For CSF monoamine adolescent studies, VFD subjects (*N* = 22) were older than non-VFD (*N* = 14) [VFD mean ± standard deviation (*SD*) = 3.44 ± 0.30 years; non-VFD mean ± *SD* = 2.85 ± 0.0.90; *t* = 2.84; *df* = 34; *p* = 0.007]. Age was therefore used as a covariate. There were 10 males and four females in the non-VFD group whereas in the VFD group there were 10 males and 12 females [χ^2^ = 2.34 (*df* = 1); *p* = 0.12]. There were no significant statistically significant effects for weight [VFD mean ± *SD* = 4.06 ± 0.61 kg; non-VFD mean ± *SD* = 3.63 ± 1.03, *t* = 1.57, *df* = 34; *p* = 0.12]. There was a trend for a rearing group × sex effect for weight [*F*_(1, 32)_ = 2.96; *p* = 0.09]. VFD females were not significantly heavier than Non-VFD females [(kg): VFD (*N* = 12) mean (*SD*) = 4.30 (0.39) vs. non-VFD (*N* = 4) mean (*SD*) = 3.77 (0.86); *df* = 14; *t*-value = 1.75; *p* = 0.11] although the effect neared a trend. VFD male weights were not distinguishable from non-VFD male weights [(kg): VFD (*N* = 10) mean (*SD*) = 3.75 (0.70) vs. non-VFD (*N* = 10) mean (*SD*) = 4.05 (0.77); *df* = 18; *t*-value = 0.88; *p* = 0.39]. Thus, the significant age difference between VFD and non-VFD did not have any significant effect on body weight even when broken down by sexes. Nevertheless, given the near trend effect for VFD females to be heavier than non-VFD females, weight was included as a covariate.

Young adult imaging data were available for 17 male macaques: 10 VFD-reared and 7 normally reared subjects. Male hippocampal volumetric data have been reported on previously (Jackowski et al., 2011). However, male and female CSF monoamine metabolite data of the current cohort have not been reported on previously. At the time of MRI scanning, there were no statistically significant differences between the groups in age [VFD mean ± *SD* = 61.20 ± 34.76 months; non-VFD mean ± *SD* = 57.63 ± 20.60; *t* = −0.24; *df* = 15; *p* = 0.81] or weight [VFD mean ± *SD* = 4.97 ± 1.08 kg; non-VFD mean ± *SD* = 4.97 ± 1.39, *t* = 0.19, *df* = 15; *p* = 0.85].

The SUNY Downstate Nonhuman Primate Facility was utilized for subject housing and the Institutional Animal Care and Use Committee of SUNY Downstate Medical Center and Mount Sinai Medical Center approved the study. Significant post-VFD experimental manipulations of either the VFD or non-VFD offspring were expressly avoided in order to prevent confounding of the VFD-rearing effects (Coplan et al., 2011).

### VFD rearing (Coplan et al., 1996)

Mother-infant dyads were group-housed in pens of 5–7 dyads each and stabilized for at least 4 weeks prior to VFD onset. After infants reached at least 2 months of age, dyads were subjected to a standard VFD procedure that involved eight alternating 2-week blocks in which maternal food was either readily accessible [low foraging demand (LFD)] or more difficult to obtain [high foraging demand (HFD)].

Foraging carts were utilized to control the difficulty in obtaining food. In the HFD condition, mothers would reach through apertures in a foraging cart to dig through clean wood-chips in order to obtain food rations. Food deprivation was avoided in the high demand condition, illustrated by normal infant weight gain and adult maintenance of baseline weights throughout the study (Dube et al., 2003). In the control (LFD) condition, there were abundant food items which the mothers picked up from the pen floor during this same 16-week period (Coplan et al., 1996). Mothers in the VFD condition undergo compromise attending to their infants' needs due to the unpredictability of foraging conditions. Achieving the early-life stress paradigm therefore occurs through the disruption of normative patterns of maternal rearing and infant attachment (Jackowski et al., 2011).

#### Housing

At one year of age, all study subjects are separated from their mothers and housed in social groups of 7–9 subjects. VFD and non-VFD-reared subject are housed separately. For the scanning procedures, males were now reproductive and were subsequently housed in single cages which permit visual, auditory, and olfactory contact with their peers.

### CSF sampling

Subjects were ushered into carrying cages from their home cages. Subjects were then released into restraint cages where they received intramuscular ketamine (15 mg/kg). Because subjects readily enter into the carrying cages, a routine procedure for weekly cleaning, physical capture of subjects was not necessary, and thus excludes the confound of “time” to capture, which has been shown to affect CSF 5-HIAA concentrations (Higley et al., 1991). Sedation of subjects was achieved in less than 5 min after exiting the carrying cage into the restraint cage. Immediately following sedation, CSF sampling was performed where one ml of CSF was taken from the cisterna magna of each monkey and immediately placed on dry ice (Kaplan et al., 1995). CSF samples were then placed in Gant tubes and stored in a −70° degree freezer. Samples were then transported to New York State Psychiatric Institute, New York, NY and stored at −70°C until assayed. The samples were used to measure concentrations of the CNS metabolites of serotonin (5-hydroxyindoleacetic acid [5-HIAA]), norepinephrine (3-methoxy-4-hydroxyphenylglycol [MHPG]), and dopamine (homovanillic acid [HVA]). The CSF monoamine metabolites were assayed using high-performance liquid chromatography with an electrochemical detector (Kaplan et al., 1995). The with-in-run and between-run coefficients of variation of the assay method were less than 10%. The level of sensitivity of this assay for 5-HIAA was 0.5 pmol/injection. Laboratory staff were blind to the rearing status of the subjects (Kaplan et al., 1995).

### Imaging

#### Scanning procedures

As described previously(Jackowski et al., 2011), on the day of the brain scan study, subjects were ushered into familiar carrying cages and transported to Mount Sinai Medical Center in a dedicated animal transport van with air-conditioning. Upon arrival at the scanner, animals were transported to a squeeze cage and following a brief restraint period, were rapidly given anesthetic agent intramuscularly. Saffan®, previously known as CT1341, is an injectable veterinary steroid anesthetic and minimizes motion artifact, relative to ketamine. Saffan®, which comprises two bioactive constituents, 12 mg/kg of alphaxalone and 4 mg/kg alphadolone acetate, was administered at a dose of 16 mg/kg. Once sedated, the monkeys' heads were positioned in a Styrofoam headrest inside a human knee coil and taped snugly over the forehead to minimize movement. Subjects remained anesthetized throughout scanning and were continuously monitored by pulse oximeter. Infrequently, if there was evidence of motion during the scan secondary to diminished level of anesthesia, animals necessitated subsequent doses of Saffan® (¼ initial dose). Subjects usually awakened within 20 min following completion of the 1 h scan. Following the imaging procedures, subjects returned on the same day to their home cages.

MRI data were acquired on a 3 T Siemens Allegra scanner. The protocol for the structural scans consisted of a three-plane sagittal localizer from which all other structural scans were prescribed. The following structural scans were acquired: axial 3D-MPRAGE (*TR* = 2500 ms, *TE* = 4.4 ms, FOV = 21 cm, matrix size = 256 × 256, 208 slices with thickness = 0.82 mm); Turbo spin echo T2-weighted Axial (*TR* = 5380 ms, *TE* = 99 ms, FOV = 18.3 × 21 cm, matrix = 512 × 448, Turbo factor = 11, 28 slices, thickness = 3 mm, skip = 1 mm).

***MRI data pre-processing and analysis***. All MRI ROI data analyses were completed by raters blind to subjects' rearing. The axial MPRAGE series were imported into ANALYZE AVW7.0 software platform. In order to isolate whole brain from its surroundings, skull, surface CSF, and meninges were stripped using a combination of tools including image thresholding, region growing, and manual tracing.

The hippocampi were manually traced using a detailed set of guidelines developed by Schumann et al. (Johansson and Roos, 1975) and adjusted, when necessary, to the bonnet macaque brain morphology using a primate brain atlas. The MPRAGEs were reoriented to the hippocampal axis, i.e., horizontal axis was parallel to a line from rostral to caudal pole of the hippocampus. The tracings were performed in oblique coronal slices, but were also checked in sagittal and axial views. Repeated measurements were performed in a random order on 5 subjects, and both intra-rater and inter-rater reliability gave an ICC of 0.93 for right/left hippocampus. An additional structural MRI data analysis was performed using VBM SPM5 (Wellcome Department of Imaging Neuroscience, London, UK; www.fil.ion.ucl.ac.uk/spm). Briefly, a single subject raised under normative conditions was chosen as the initial template. All images were registered through linear (zooms, rotations, translations, and shears) deformations to the single subject template. An average image, called template henceforth, was created with the obtained deformed images. Afterwards, the same original images were linearly deformed to the created template and this step was iterated 20 times to minimize the bias caused by utilization of a single subject as the initial template. On the 22nd step, original images were linearly and also non-linearly registered to the final template. A brain mask containing gray matter, white matter and CSF, was manually delineated for the template and used to eliminate skull and meninges from the final registered images. In order to preserve brain volume, images were scaled using the Jacobian matrix, so that the total amount of gray matter in the resulting images remains the same as it would be in the original images. The obtained images were finally smoothed with a Gaussian filter at Full Width at Height Maximum equal to 4 mm (Jackowski et al., 2011).

DTI (Coplan et al., 2010) used a pulsed-gradient spin-echo sequence with EPI-acquisition (*TR* = 4100 ms, *TE* = 80 ms, FOV = 21 cm, matrix = 128 × 128, 24 slices, thickness = 3 mm skip 1 mm, b-factor = 1250 s/mm^2^, 12 gradient directions, 5 averages). Raw DTI data were transferred to an off-line workstation for post-processing. In-house software written in Matlab v6.5 (The Mathworks Inc. Natick, MA) was used to compute the anisotropy and vector maps. FA images were then converted to analyze format. MEDx v3.4.3 software (Medical Numerics Inc, Sterling, VA) was used to inspect and define ROIs on the FA images. Regions of interest (ROI) included the left and right anterior limb of the internal capsule (ALIC).

### Statistical analysis

CSF monoamines and hippocampal volume values were examined to assure normal distribution using the Kolmogorov-Smirnov test and Lilliefors test. Evidence of non-normal distributions were to be followed by non-parametric testing. Scatterplots of variables were examined for outliers and excluded *a priore*.

***CSF monoamine rearing group analyses***. As the age of VFD subjects was significantly elevated relative to non-VFD subjects for CSF monoamine analyses, age was used as a covariate for between-rearing group comparisons of monoamine metabolites. Pearson's correlations were used to examine for age effects on monoamines in males and females combined, males alone and females alone. Sex was also applied as a covariate *a priore* as sex effects for CSF 5-HIAA have previously been demonstrated in other non-human primate species (Higley et al., 1991). Thereafter, body mass was to be used as a covariate as a proxy for pubertal status. Pearson's correlations were used to examine for weight effects on monoamines in males and females combined, males alone and females alone.

***CSF monoamine concentrations and hippocampal volume***. For examination of the relationship between CSF monoamines and hippocampal volume, since all subjects who had imaging data were male, controlling for sex effects were not necessary. Female scans have been performed in a follow-up study and are currently being analyzed. In the non-VFD, one male subject was excluded because their right hippocampal volume of 0.57 mm^3^ was 3.75 *SD*s above the group mean of 0.45 ± 0.032 mm^3^ (*N* = 6). There were no significant relationships between the volume of total brain, left hippocampus and right hippocampus, and with any of the CSF monoamine metabolites, thus brain volume was not used as a covariate. Young adult age and weight were also not used as covariates as they were not associated with hippocampal volume or CSF monoamine metabolite levels. Within-rearing group Pearson's correlations were determined to test the hypothesis, outlined in the introduction, that monoamine metabolite concentrations, particularly CSF 5-HIAA, would be inversely related to hippocampal volume, particularly in VFD-reared subjects for whom early life stress was posited to accentuate the inverse relationship. For determination of the predictive value of rearing effects in the induction of the putative inverse relationship between hippocampal volume and each CSF monoamine metabolite concentration, general linear model (GLM) analyses were conducted separately using left and right hippocampal volume as the continuous predictor variable. Rearing group was used as a categorical independent variable and CSF monoamine metabolite as the dependent variable. The factorial interaction term of right or left hippocampal volume × rearing condition was also entered into the GLM to examine whether the relationship between hippocampal volume and each CSF monoamine metabolite differed as a function of ELS. The rationale of using both left and right hippocampal volume as independent predictor variables in separate GLM's is that VFD-rearing has previously been shown, in the current cohort, to be associated with reduced left, but not right, hippocampal volume in comparison to non-VFD subjects (Jackowski et al., 2011).

***CSF monoamine concentrations and fractional anisotropy (FA) of the ALIC***. We examined the relationship between CSF monoamines and white matter FA of the ALIC. We have previously documented reduced FA in the ALIC bilaterally following ELS (Coplan et al., 2010). Thus, mean FA [(right plus left)/2] was used as the continuous predictor variable in an identical GLM to that outlined for hippocampal volume. The underlying hypothesis outlined in the introduction was that CSF 5-HIAA concentrations would associate inversely with FA of ALIC and the association would be specific to VFD subjects.

In summary, the primary hypothesis was to detect elevated CSF 5-HIAA in VFD vs. non-VFD. The second hypothesis was to demonstrate an inverse correlation between CSF 5-HIAA concentrations and right hippocampal volume in VFD. The third hypothesis was to demonstrate an inverse relationship between CSF 5-HIAA and FA of the ALIC in VFD. Since each hypothesis was independent, significance was set at *p* ≤ 0.05, two tailed.

### Results

#### CSF monoamine analysis

A normal distribution of CSF MHPG [Kolmogorov-Smirnoff (K-S) D = 0.07, *p* > 0.20; Lilliefors *p* > 0.20], CSF HVA [(K-S) D = 0.14, *p* > 0.20; Lilliefors *p* < 0.10] but not CSF 5-HIAA [(K-S) D = 0.25, *p* < 0.05; Lilliefors *p* < 0.01] was observed. Inspection of the histogram revealed an extended distribution on the right tail. Even though CSF 5-HIAA was both log^10^ and square-root transformed, distribution still remained non-normal. Therefore, parametric analyses, where age, weight or sex could be used as covariates, were performed followed by the non-parametric Mann-Whiney U. No outliers were noted for the combined male and female analyses.

***Age effects***. There was no sex effect for age [years: males (*N* = 20) mean (*SD*) = 3.20 (0.71) vs. females (*N* = 16) mean (*SD*) = 3.20 (0.61); *t*-value = −0.006; *df* = 34; *p* = 0.95]. There was no effect of age on CSF 5-HIAA (*r* = 0.18; *N* = 36; *p* = 0.18), CSF HVA (*r* = −0.05, *N* = 36; *p* = 0.76) but there was a significant inverse effect for CSF MHPG (*r* = −0.37; *N* = 36; *p* = 0.025). There were no age effects in males for CSF 5-HIAA or HVA (*N* = 20; all *p*-values > 0.37) but there was an inverse effect for CSF MHPG (*r* = −0.47; *p* = 0.34). There were no age effects for females (*N* = 16; all *p* values > 0.46). There were no sex × age effects for CSF 5-HIAA [*F*_(1, 32)_ = 0.00; *p* = 0.92].

***Weight effects***. There was no sex effect for weight [kg: males (*N* = 20) mean (*SD*) = 3.90 (0.73) vs. females (*N* = 16) mean (*SD*) = 4.17 (0.56); *t*-value = 1.23; *df* = 34; *p* = 0.22]. When males and females were combined, body weight bore no significant relationship to CSF 5-HIAA (*r* = 0.17; *N* = 36; *p* = 0.30), HVA (*r* = 0.02; *N* = 36; *p* = 0.88) or MHPG (*r* = −0.18; *N* = 36; *p* = 0.28). There were no weight effects for CSF monoamines in males (*N* = 20; all *p* values > 0.43) or females (*N* = 16, all *p* values > 0.38). There were no sex × weight effects in the prediction of CSF 5-HIAA [*F*_(1, 32)_ = 0.37; *p* = 0.54].

**Figure 1:**
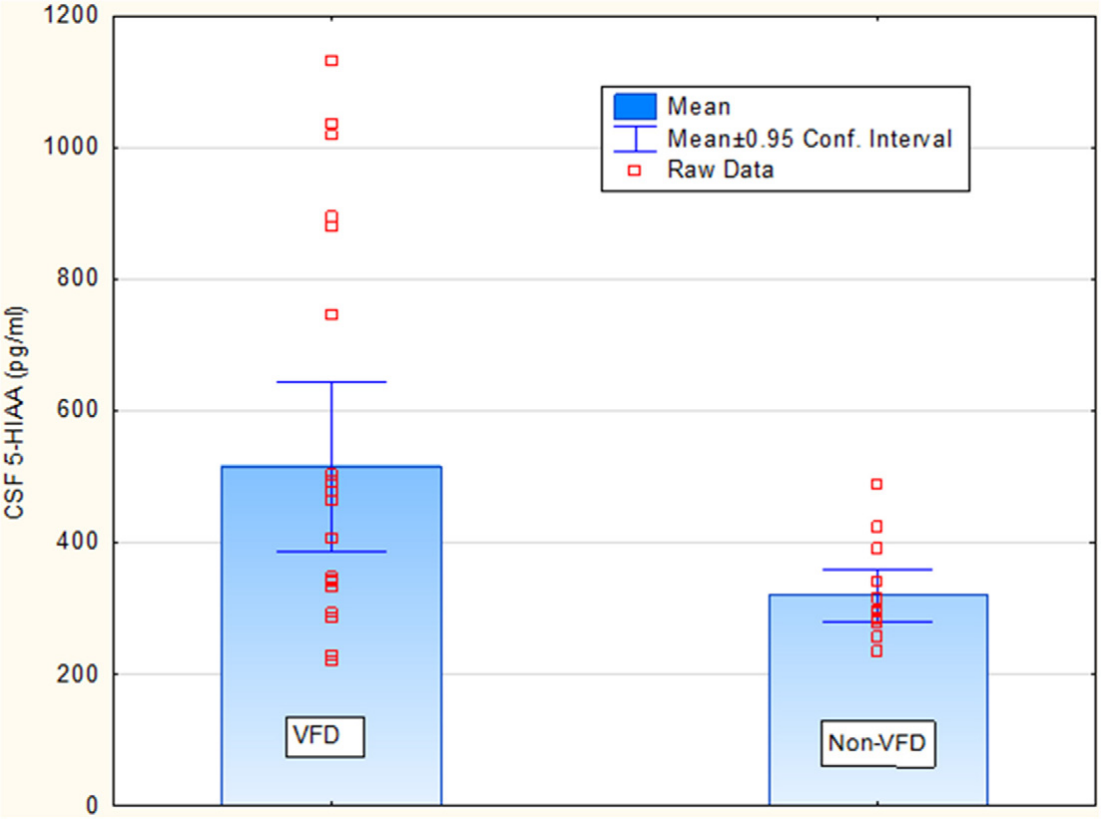
**Rearing group differences for cisternal CSF 5-HIAA concentrations in non-human primates**. Covarying for age, [*F*_(1, 33)_ = 0.00; *p* = 0.93], VFD-reared subjects exhibited elevated CSF 5-HIAA concentrations (ng/ml) relative to non-VFD-reared counterparts [VFD mean (*SE*) = 515.40 (50.50) (*N* = 22) vs. non-VFD mean (*SE*) = 319.60 (63.30), *N* = 14; *F*_(1, 33)_ = 4.56; *p* = 0.04] whereas no group effects were observed for CSF MHPG and CSF HVA concentrations (*p* > 0.1). The CSF 5-HIAA finding was confirmed using the non-parametric Mann Whitney U (*Z* = 2.28; *p* = 0.022).

Covarying for age, [*F*_(1, 33)_ = 0.00; *p* = 0.93], VFD-reared subjects exhibited elevated CSF 5-HIAA concentrations (ng/ml) relative to non-VFD-reared counterparts [VFD mean (*SE*) = 515.40 (50.50) (*N* = 22) vs. non-VFD mean (*SE*) = 319.60 (63.30), *N* = 14; *F*_(1, 33)_ = 4.56; *p* = 0.04] whereas no group effects were observed for CSF MHPG and CSF HVA concentrations (*p* > 0.1). Covarying for sex, CSF 5-HIAA remained elevated in VFD-reared subjects vs. controls [*F*_(1, 33)_ = 5.60; *p* = 0.023]. Covarying for weight, CSF 5-HIAA remained elevated in VFD-reared subjects vs. controls [*F*_(1, 33)_ = 5.68; *p* = 0.022]. The CSF 5-HIAA finding was confirmed using the non-parametric Mann Whitney U (*Z* = 2.28; *p* = 0.022) (Figure 1). Inspection of the CSF HVA scatterplot revealed a cut-off of 1600 pg/ml for which an excess of VFD values were evident. Nine of 22 VFD whereas 1/14 non-VFD subjects fell above the 1600 pg/ml cut-off (Fisher exact *p*, one-tailed *p* = 0.029) providing modest support for an elevation of CSF HVA in VFD-reared subjects. No sex effects or sex by rearing group effects were noted for any of the CSF monoamines.

#### Relationship of CSF monoamines to hippocampal volume

In a Pearson's correlation matrix, significant inverse correlations were observed in the VFD but not non-VFD group for right hippocampus and each of the CSF monoamine metabolite (see Table 1). All other correlations were non-significant.

**Table 1:** **Correlations between hippocampal volume and CSF monoamine metabolites as a function of early life stress**.

**CSF monoamine metabolite**	**VFD = 10**		Non-VFD = 6^*^	
**Side**	**Right**	**Left**	**Right**	**Left**
5-HIAA (pg/ml)	**−0.65**	−0.01	0.49	0.75
	***p*** = **0.041**	*p* = 0.97	*p* = 0.31	*p* = 0.084
MHPG (pg/ml)	**−0.74**	−0.41	0.43	0.68
	***p* = 0.013**	*p* = 0.22	*p* = 0.38	*p* = 0.13
HVA (pg/ml)	**−0.85**	−0.23	0.38	0.72
	***p* = 0.002**	*p* = 0.50	*p* = 0.45	*p* = 0.10

* *One outlier excluded. CSF, cerebrospinal fluid; 5-HIAA, 5-hydroxyindoleacetic acid; MHPG, 3-methoxy-4-hydroxyphenylglycol; HVA, homovanillic acid*.

**Figure 2:**
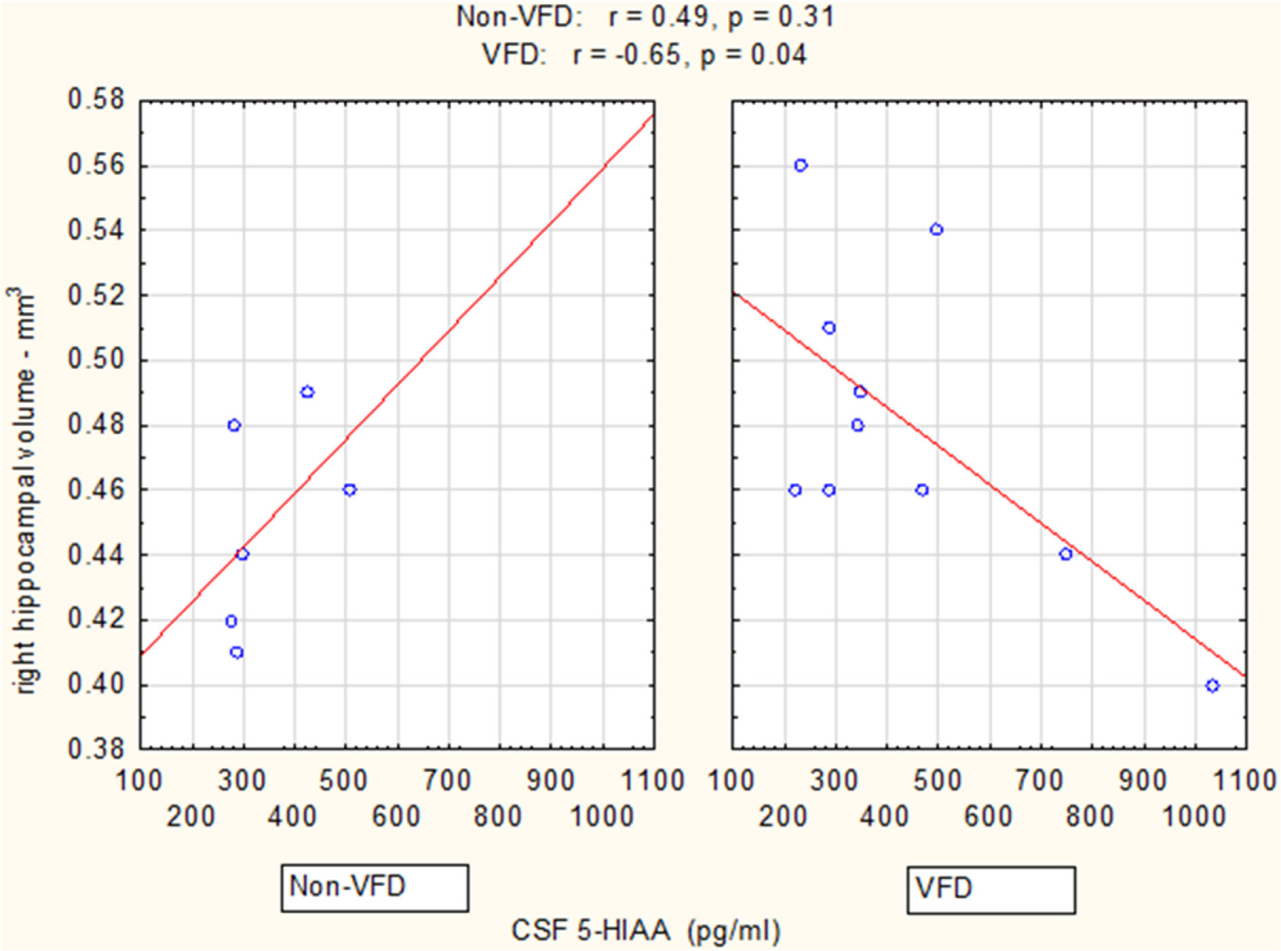
**Relationship between right hippocampal volume and CSF 5-HIAA as a function of early life stress**. For CSF 5-HIAA, right hippocampus correlated inversely in VFD-reared subjects and but not in non-VFD subjects [right hippocampal × rearing condition interaction: *F*_(1, 12)_ = 3.29, *p* = 0.095]. See Pearson's correlations at the top of the figure.

**Figure 3:**
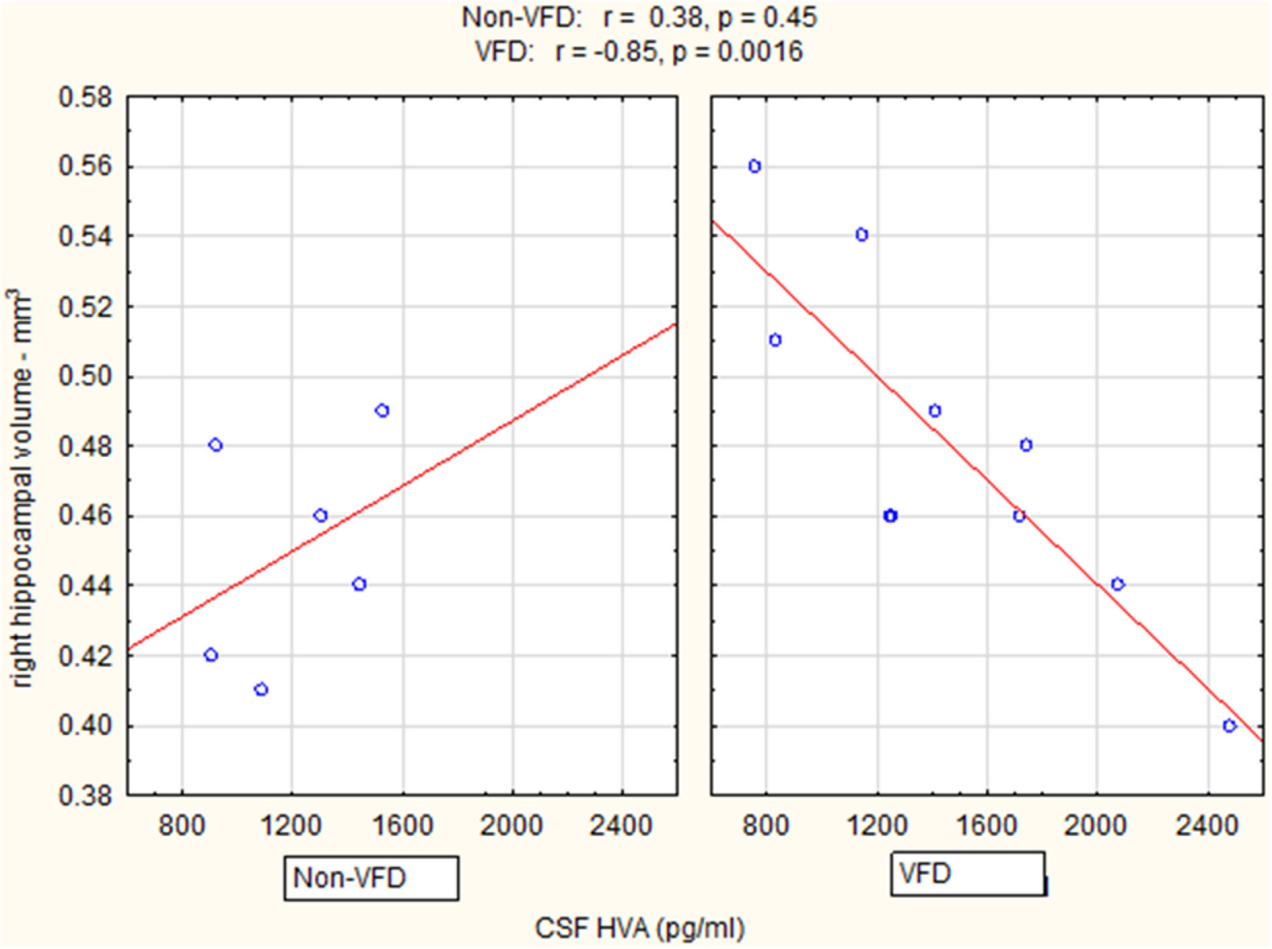
**Relationship between right hippocampal volume and CSF HVA as a function of early life stress**. For CSF HVA, right hippocampus correlated inversely in VFD-reared subjects and but not in non-VFD subjects [right hippocampal × rearing condition interaction: *F*_(1, 12)_ = 8.22, *p* = 0.014]. See Pearson's correlations at the top of the figure.

**Figure 4:**
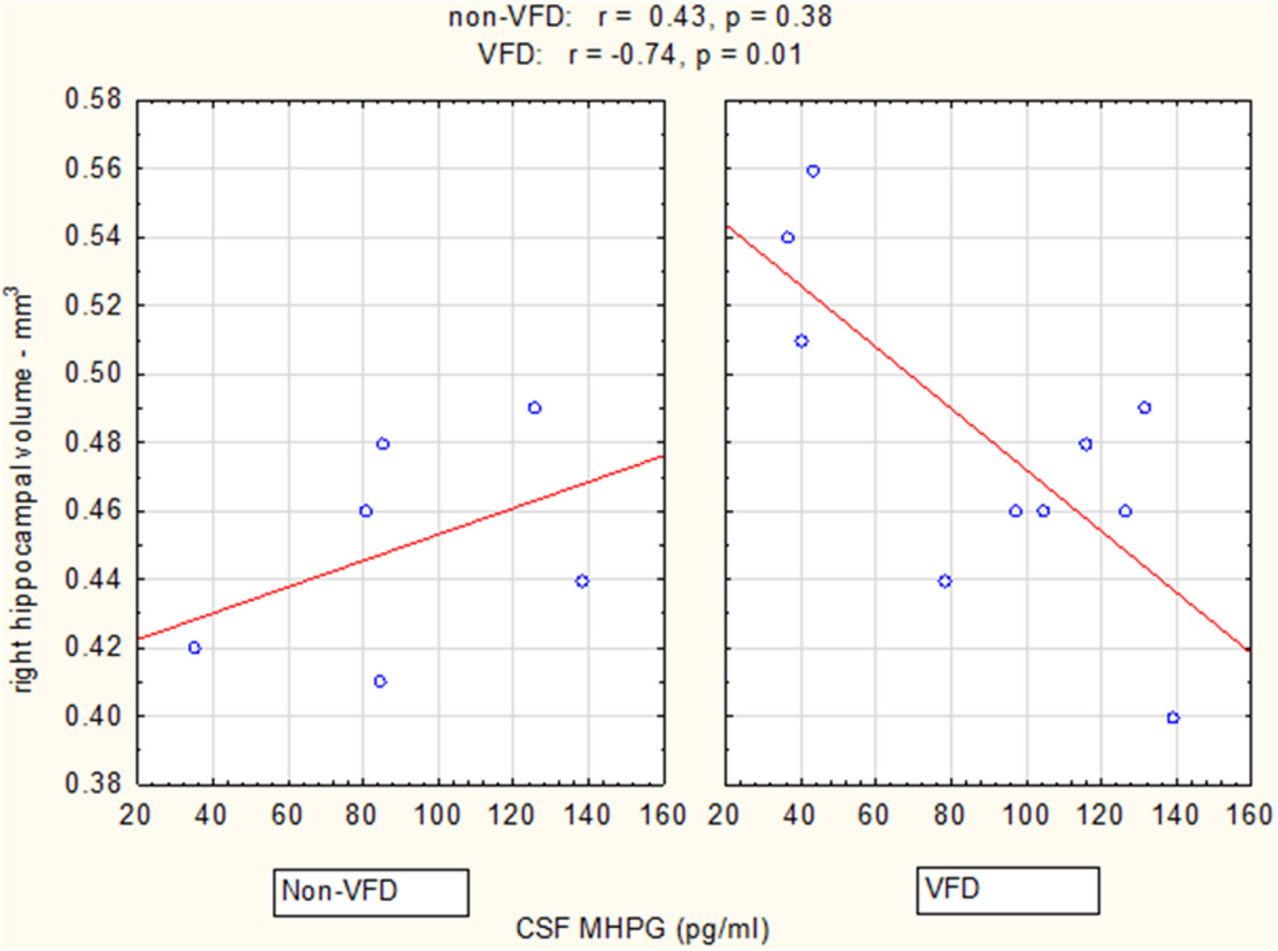
**Relationship between right hippocampal volume and CSF MHPG as a function of early life stress**. For CSF MHPG, right hippocampus correlated inversely in VFD-reared subjects and but not in non-VFD subjects [right hippocampal × rearing condition interaction: *F*_(1, 12)_ = 5.37, *p* = 0.039]. See Pearson's correlations at the top of the figure.

Controlling for right hippocampal volume, CSF 5-HIAA was marginally elevated in VFD vs. non-VFD [VFD mean (*SE*) = 446.19 (56.58) *N* = 10 vs. non-VFD mean (*SE*) = 344.94 (73.05) *N* = 6; *F*_(1, 12)_ = 3.79; *p* = 0.075], CSF HVA was elevated in VFD [VFD mean (*SE*) = 1468.38 (92.06) *N* = 10 vs. non-VFD mean (*SE*) = 1200.31 (118.85) *N* = 6; *F*_(1, 12)_ = 9.56; *p* = 0.009], whereas MHPG was slightly yet significantly diminished in VFD [VFD mean (*SE*) = 91.17 (9.85) *N* = 10 vs. non-VFD mean (*SE*) = 91.61 (12.71) *N* = 6; *F*_(1, 12)_ = 5.57; *p* = 0.036]. For CSF HVA, a right hippocampal volume × rearing effect was observed [*F*_(1, 12)_ = 8.22, *p* = 0.014, see Figure 3, Table 1] as the two variables correlated inversely in VFD-reared subjects and but not in non-VFD subjects. A similar right hippocampal volume × rearing effect was observed for CSF MHPG [*F*_(1, 12)_ = 5.37, *p* = 0.039, see Figure 4, Table 1]. The right hippocampal volume × rearing interaction effect for CSF 5-HIAA was only significant at a trend level [*F*_(1, 12)_ = 3.29, *p* = 0.095, see Figure 2, Table 1]. Right hippocampal volume was not associated with any CSF monoamine when the groups are considered in a combined fashion. No effects for the GLM were observed when using left hippocampus as a predictor variable.

#### Relationship of CSF monoamines to white matter FA of the ALIC

**Figure 5:**
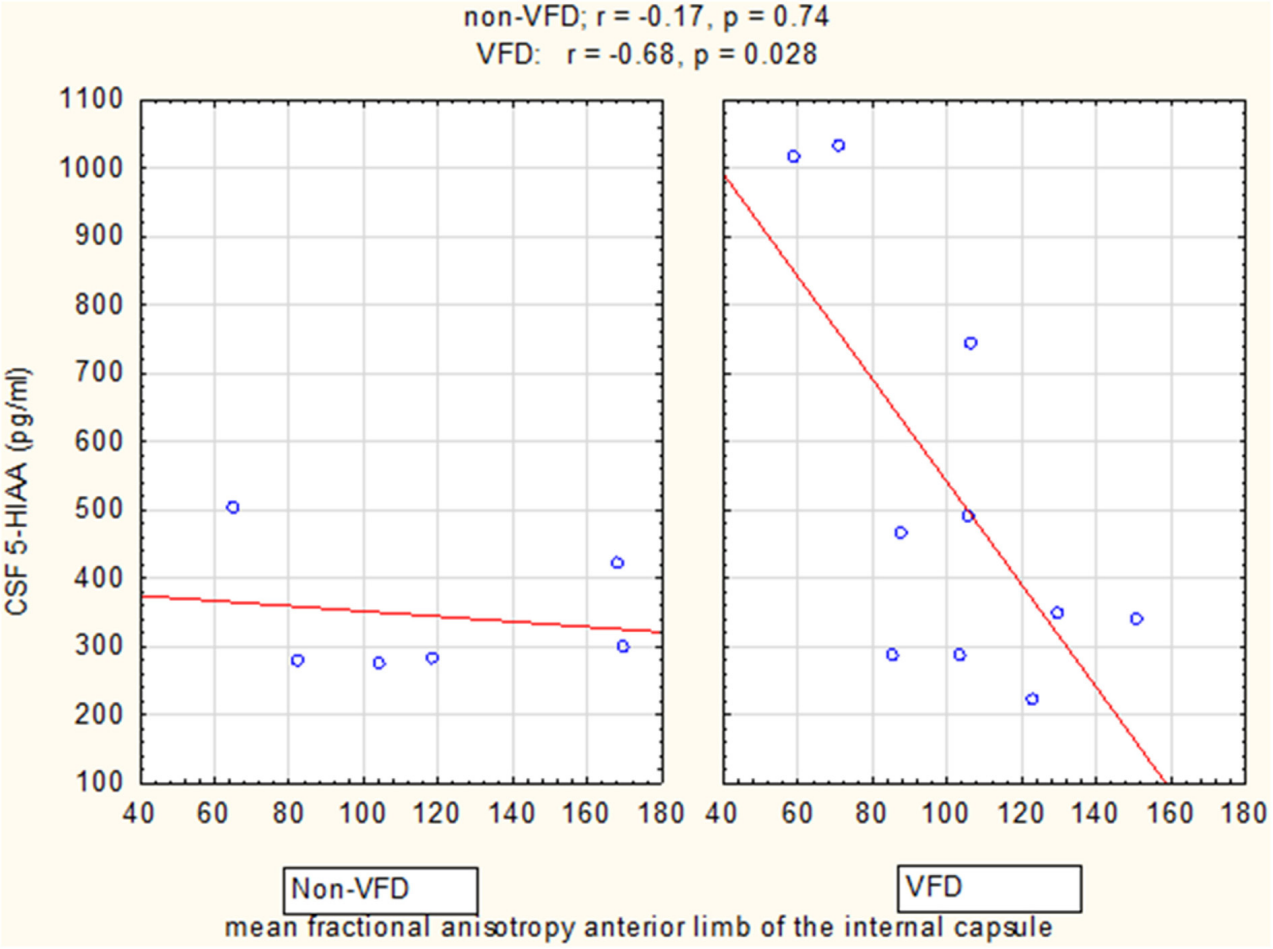
**Relationship of cisternal CSF 5-HIAA concentrations to mean fractional anisotropy of the anterior limb of the internal capsule**. For mean fractional anisotropy (FA) of the anterior limb of the internal capsule (ALIC), CSF 5-HIAA correlated inversely in VFD-reared subjects and but not in non-VFD subjects [rearing group × mean FA interaction: *F*_(1, 12)_ = 5.02, *p* = 0.044]. See Pearson's correlations at the top of the figure.

GLM analyses revealed a significant group effect only for CSF 5-HIAA [VFD mean (*SE*) = 525.14 (63.39) *N* = 10 vs. non-VFD mean (*SE*) = 344.94 (81.84) *N* = 6; *F*_(1, 12)_ = 5.09, *p* = 0.04], adjusted for mean FA of the ALIC (Figure 5). Mean CSF 5-HIAA was overall inversely associated with FA of the anterior limb of the internal capsule [*F*_(1, 12)_ = 6.16; *p* = 0.028]. A significant rearing group × mean FA interaction predicting CSF 5-HIAA was noted [*F*_(1, 12)_ = 5.02; *p* = 0.044]. The Pearson's correlation between CSF 5-HIAA and mean ALIC FA was inversely significant in VFD [*r* = −0.68; *N* = 10; *p* = 0.029] but not in non-VFD subjects [*r* = −0.17; *N* = 6; *p* = 0.74]. A similar group effect was noted for CSF HVA concentrations on GLM analysis [*F*_(1, 12)_ = 4.70, *p* = 0.05] when controlling for mean FA of ALIC, but no other CSF HVA effects were noted. No effects were noted for CSF MHPG.

## Discussion

The current study replicates our finding of elevated cisternal CSF 5-HIAA in two previous cohorts of macaque subjects reared under the VFD model of ELS (Mendels and Frazer, 1974; Coplan et al., 1998). The data indicate that maternal uncertainty produced by variable foraging conditions may render offspring vulnerable to developing abnormally elevated levels of CSF 5-HIAA (Mendels and Frazer, 1974; Coplan et al., 1998). Effects are independent of age, weight and sex, which is consistent with our previous studies (Coplan et al., 1996; Mathew et al., 2002) but in contrast to rhesus studies, where age, pubertal status and sex have been demonstrated to influence CSF 5-HIAA concentrations (Higley et al., 1991). In this VFD model of ELS, we find that putatively high peri-raphe concentrations of serotonin are correlated with markers of reduced neurotrophic activity in structures affected by serotonin pathways, specifically the right hippocampus, suggesting that high cisternal CSF 5-HIAA is associated with attenuated neurotrophic effects.

To date, lumbar CSF 5-HIAA measures to characterize the status of serotonin neurotransmission in mood disorders, with the exception of suicidal patients, have reported inconsistent findings (Bowers et al., 1969; Papeschi and McClure, 1971; Mendels and Frazer, 1974). These inconsistencies may stem, in part, from sampling of CSF via lumbar puncture. Lumbar CSF may reflect an aggregate of spinal, midbrain, frontal, and limbic projection-site 5-HIAA concentrations, therefore failing to identify monoamine metabolite measures in specific localized brain regions. The concentration gradient of monoamine metabolites in lumbar CSF means levels vary with the amount of fluid taken (Johansson and Roos, 1975; Sjöström et al., 1975), suggesting differences in regional concentrations. In macaques, cisternal CSF is usually drawn from the cerebello-pontine angle via an atlanto-occipital route reasonably adjacent to the midbrain, which may preferentially reflect diffusion from peri-raphe extracellular fluid. Regional differences, however, in CSF 5-HIAA concentrations have not, to our knowledge, been systematically reported, and CSF 5-HIAA may represent a homogenous pooled resource coming from multiple areas of the brain. For instance, Ichise et al. (2006) reported a strong correlation between serotonin transporter binding and cisternal CSF 5-HIAA concentrations in a number of areas of the brain, but interestingly did not find a correlation between transporter binding in the raphe and CSF 5-HIAA concentrations.

Our model, however, is in accordance with a recent pilot study of suicides that found high levels of 5-HIAA in the midbrain (and was also associated with reduced 5-HIAA in projection tissues such as the PFC) (Bach et al., 2014). In past studies, we have demonstrated that VFD subjects exhibit elevated CSF corticotropin-releasing factor (CRF) measures and these CSF CRF concentration elevations correlated directly with CSF 5-HIAA concentrations only in VFD-reared subjects (Coplan et al., 1996). These heightened levels of CRF reveal a hyperactive stress-response system in the VFD brain (Holmes et al., 2003a), a scenario found to persist long term in rodents subjected to early life stress and human patients with a perception of early adversity (Liu and Weiss, 2002; Holmes et al., 2003a; Carpenter et al., 2004; Huot et al., 2004; Ladd et al., 2004; Brunson et al., 2005). The current findings are intriguing given growing evidence of a close inter-relationship between CRF and serotonin in mediating stress responses (Roche et al., 2003). Recent data show that both stress and CRF release attenuate serotonin neurotransmission in specific forebrain structures, suggesting that stress may activate CRF-mediated inhibitory afferents to the DRN, decreasing serotonin release to the forebrain (Roche et al., 2003). In addition, excitatory glutamate-mediated mPFC afferents stimulate serotonin release in the DRN in response to stress, in turn activating a negative feedback mechanism via inhibitory somatodendritic 5-HT_1A_ autoreceptors located on serotonergic neurons in the DRN, attenuating serotonin neurotransmission (Blier et al., 1998; Celada et al., 2001; Valentino et al., 2003). Adult knockout mice lacking the gene for the serotonin transporter protein (Brunson et al., 2005) exhibit increased concentrations of peri-raphe serotonin compared to wild-type mice, providing a useful model for how the serotonin system functions under conditions of high dorsal raphe serotonin. In these subjects, the dorsal raphe serotonergic neurons are in a perpetual state of hyperpolarization (Lira et al., 2003), with diminished synaptic serotonin at terminal projection sites, such as the hippocampus. Behaviorally, both depression-like and anxious behaviors were observed in these serotonin transporter knockout mice (Lira et al., 2003; Holmes et al., 2003b). Taken together, the data bolster the plausibility that elevated cisternal CSF 5-HIAA may in fact be indicative of a serotonin deficit downstream of the raphe nucleus, but the study was not designed to document this putative serotonin deficit. Moreover, we have hypothesized elsewhere that elevations of midbrain serotonin would counteract the efficacy of SSRI's, as appreciable 5-HT_1A_ autoreceptor downmodulation, essential for SSRI efficacy (Blier et al., 1987), would no longer be feasible (Coplan et al., 2014).

In the current study, we found that relatively high VFD cisternal 5-HIAA measures correlated with reduced right hippocampal volume, a potential index of neurotrophic activity. Hippocampal volume is closely reliant on the presence of neurotrophic factors such as brain-derived growth factor (BDNF) to maintain neuronal growth and survival (Duman et al., 2000). The production of BDNF and other similar factors is instigated by serotonin-initiated cellular messenger signal proteins, implying that reduced serotonin neurotransmission at the hippocampus may conceivably cause reduced volume (Duman et al., 2000; Daniels et al., 2013). Other signaling pathways may also increase BDNF such as peripheral growth factors that are increased by physical exercise (Cotman et al., 2007). BDNF, nevertheless, has an important synergistic relationship with the serotonin system, regulating serotonergic neuronal survival while serotonin-dependent second messengers modulate BDNF production (Martinowich and Lu, 2007). The hippocampus is particularly vulnerable to changes in BDNF level; missense polymorphisms in the BDNF gene are directly correlated with reduced hippocampal volume (Szeszko et al., 2005).

That reduction of FA measures of white matter integrity in the ALIC inversely associated with elevated cisternal CSF 5-HIAA provide convergent validation of neurotrophic compromise secondary to a putative serotonin deficit state in downstream structures of the serotonergic pathway. Parental verbal abuse is linked to a decrease in FA in a variety of white matter tracts (Choi et al., 2009), indicating there is a link between ELS and WM integrity. A number of studies illustrate the influence of serotonin on WM integrity. Variations in WM integrity appear to be regulated by serotonin transporter genotype in rhesus monkeys (Howell et al., 2014), while patient response to SSRIs is predicted by FA measures (DeLorenzo et al., 2013). Intracellularly, the serotonin-dependent neurotrophin, BDNF, enhances WM integrity by increasing concentrations of myelin basic protein and facilitating oligodendrocyte proliferation (McTigue et al., 2001; Wong et al., 2013). The current study reveals that relatively elevated cisternal CSF 5-HIAA is associated with reduced FA in the ALIC of VFD-reared subjects. We acknowledge that other areas of white matter in addition to the anterior limb of the internal capsule such as the fornix, which is intimately related to the hippocampus (Kuroki et al., 2006), should have been considered for study. We contend that the choice of the ALIC is justified, not only because reduced FA in the ALIC has been found in a range of psychiatric disorders (Kubicki et al., 2005; Zhang et al., 2013) but that our contention is that the high cisternal CSF 5-HIAA compromises serotonin-related function across a range of target projection site, and that the ALIC provides a useful WM proxy of neurotrophic compromise. Further supporting our results is the finding of an inverse relationship between the dopamine and norepinephrine metabolites—HVA and MHPG, with hippocampal volume in VFD subjects. These associations are statistically absent in non-VFD subjects. CSF HVA and 5-HIAA levels consistently show a close correlation (Papeschi and McClure, 1971). With respect to MHPG, we have previously reported that in patients with panic disorder, enhanced serotonin neurotransmission by SSRI's is associated with lowered plasma MHPG concentrations (Coplan et al., 1997). In the noradrenergic locus ceruleus (LC), serotonin attenuates neuronal response to glutamate, a major neurotransmitter utilized by afferents to the LC (Aston-Jones et al., 1991). In the VFD midbrain, elevated cisternal CSF 5-HIAA, acting via inhibitory 5-HT_1A_ autoreceptors, may conceivably attenuate serotonin outflow, thereby reducing serotonin-mediated inhibition of LC neuronal firing (Aston-Jones et al., 1991). In this putative serotonin deficit state, paradoxically high CSF 5-HIAA would be positively associated with CSF MHPG. Thus, inverse associations between cisternal CSF HVA and MHPG concentrations and right hippocampal volume serve as proxy indicators of the relation between cisternal CSF 5-HIAA and hippocampal volume, specifically in subjects reared under conditions of ELS.

Although our findings seemingly conflict with the CSF 5-HIAA deficit consistently observed in maternally-separated peer-reared vs. maternally-reared rhesus monkeys (Higley et al., 1991), it is important to note that the low cisternal CSF 5-HIAA associated with peer rearing is linked not only to affective distress (Clarke and Snipes, 1998) but also impulsivity and aggression (Higley et al., 1991; Higley and Linnoila, 1997), whereas VFD subjects exhibit consistent disturbances in affective domains without impulsivity and aggression (Rosenblum and Paully, 1984; Higley et al., 1991; Rosenblum et al., 2001). Thus, the two forms of ELS—maternal deprivation vs. maternal uncertainty—result in divergent CSF 5-HIAA findings and, despite overlap in the affective domain, divergence in impulsive and aggressive behavior.

Moreover, in vervet monkeys, reduced cisternal CSF 5HIAA was also found to be inversely associated with social impulsivity (Fairbanks et al., 2001). By contrast, subordinate female rhesus, a chronic form of unpredictable stress, is associated with high CSF 5-HIAA (Asher et al., 2013). The data imply that specific forms of ELS may induce specific variations in serotonin neurotransmission. A range of distinct neurobiological sequelae in response to various patterns of ELS have therefore been reported (Mathew et al., 2001).

Limitations of the present study include the restriction of the imaging data to a subset of the males in the sample although the CSF 5-HIAA elevations following ELS were not sex-specific. The inverse association between CSF 5-HIAA, MHPG, and HVA in VFD-reared subjects and hippocampal volumes was restricted to the right side. It is interesting to note that we have previously reported in this same cohort reduced left hippocampal volume in VFD-reared vs. non-VFD-reared controls whereas differences for right hippocampal volume were not significant (Jackowski et al., 2011). It is conceivable that the left hippocampal volume has been reduced by ELS to an extent that a “floor” effect is in place. Thus, left hippocampus is not sufficiently intact to be affected by altered ranges of serotonin neurotransmission. A major limitation of the paper is that a serotonin deficit state in target projection areas can only be inferred and was not directly documented. Future post-mortem studies on VFD vs. control subjects may provide a methodology whereby a serotonin-deficit state in VFD hippocampus can be definitively ascertained. Nevertheless, precedent for high peri-raphe serotonin and/or its primary metabolite, 5-HIAA, and low PFC serotonin/5-HIAA is present in suicides (Bach et al., 2014). Moreover, a preclinical precedent for low hippocampal serotonin in the face of high peri-raphe serotonin is observed in serotonin transporter knockout mice (Lira et al., 2003). Moreover, the acute effects of serotonin transporter blockade by SSRIs is to increase peri-raphe serotonin, reduce serotonin neuronal firing and reduce distal synaptic release, negating immediate antidepressant effects of the SSRIs (Haddjeri et al., 1998). Despite cogent precedents of high peri-raphe serotonin leading to reduced serotonin outflow, direct documentation of a hippocampal serotonin deficit state in VFD subjects with high cisternal CSF 5-HIAA is required in order to draw a definitive conclusion. Although we have posited that cisternal CSF is a preferable site to document alterations of serotonin neurotransmission, going forward, direct quantification on post-mortem samples of non-human primates would be required to provide definitive validation of the view that ELS effects an increase in midbrain serotonin, thereby inducing a serotonin deficit state in terminal fields. For instance, Knott et al. (1989), reported a positive relationship between ventricular 5-HIAA and cortical 5-HIAA in post-mortem brain of patients with Alzheimer's disease, emphasizing the need for regional quantification. The use of the serotonin metabolite, 5-HIAA, requires validation as a marker of 5-HT. Increased turnover, which may reduce serotonin and increase 5-HIAA, should be ruled out by direct measurement of serotonin. A further limitation of the study is the age difference between the VFD-reared vs. normally-reared controls. Although this difference is only in the order of six months, one primate year is approximately 3–4 human years. This would suggest that the 6 month difference between VFD and controls of 6 months could be an 18 months to two year difference in humans. However, despite this limitation, using age as a covariate did not affect the significance of the results. Furthermore, age was not related to CSF 5-HIAA, either when considering male and females combined, males alone or females alone. In addition, the subjects in the current study were at an age where they were peripubertal (Kaufman and Rosenblum, 1966). Puberty has been noted to affect CSF 5-HIAA in rhesus (Higley et al., 1991). Lack of measurement of gonadal hormones prevents covariation for the confounding effects of pubertal status. Furthermore, since females enter puberty before males, the fact that the VFD group were 6 months older than the non-VFD could further compound the confound introduced by gonadal hormones. Using weight as a proxy for pubertal growth, we noted that there were no weight effects on CSF 5-HIAA, either when considering male and females combined, males alone or females alone. Moreover, there were no sex effects for weight and there was a trend for a rearing group × sex effect for body weight. *Post-hoc t*-tests revealed that VFD females were not heavier than non-VFD females but because this comparison was a near trend, weight was used as a covariate when assessing rearing group differences for CSF 5-HIAA. Elevations in CSF 5-HIAA concentrations in VFD compared to non-VFD subjects remained significant. Finally, the inverse relationship between CSF 5-HIAA and right hippocampal volume is correlational but not causal. Other factors, such as HPA axis alterations, may represent an intermediary step between alterations in serotonin function and hippocampal volume. Moreover, without causal evidence, the directionality of the hypothesis stemming from altered serotonin function to adversely affect hippocampal remains an assumption. Evidence for the hypothesis, to the extent supported by convergent processes, is provided by the inverse relationship between CSF 5-HIAA concentrations and white matter FA. Studies directed at confirming the causality of CSF 5-HIAA elevations in the pathophysiology of hippocampal volume reductions are warranted.

### Conflict of interest statement

Dr. Mann receives royalties for commercial use of the C-SSRS from the Research Foundation for Mental Hygiene and has stock options in Qualitas Health, a start-up company developing a PUFA food supplement. Dr. Mathew has been named as an inventor on a pending use-patent of ketamine for the treatment of depression. Dr. Mathew has relinquished his claim to any royalties and will not benefit financially if ketamine were approved for this use. Dr. Mathew has received consulting fees. The authors declare that the research was conducted in the absence of any commercial or financial relationships that could be construed as a potential conflict of interest.
